# Supplementary material for: Wetter summers can intensify departures from natural variability in a warming climate
Source: Nat Commun. 2018 Feb 22;9:783. doi: 10.1038/s41467-018-03132-z (PMC5823852; doi:10.1038/s41467-018-03132-z)
Supplement: Supplementary file 1 — Supplementary Information [file 41467_2018_3132_MOESM1_ESM.pdf]

Supplementary Notes

Supplementary Note 1: Relative departures of temperature and precipitation

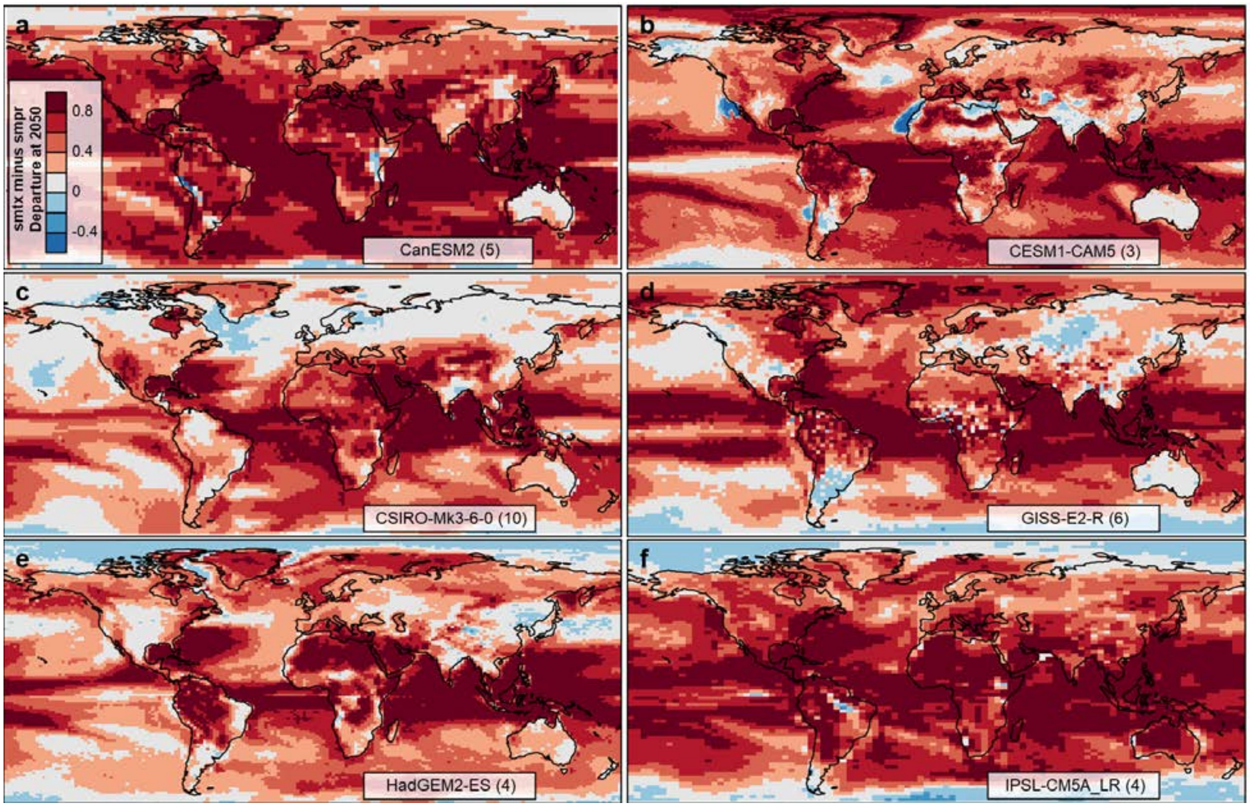

Supplementary Figure 1 | Relative departures (2σ proportions) of summer precipitation (smpr) and mean daily maximum temperature in the summer (smtx) in CMIP5 projections of the 2021-2050 period. Negative values (blue) indicate locations where the smpr departure is greater than the smtx departure. Panels a-f show single-model ensemble results for each of six CMIP5 models. The number of runs in each ensemble is given in parentheses next to the model name.

Supplementary Note 2: Summer Tx-Pr correlations and climate change orthogonality

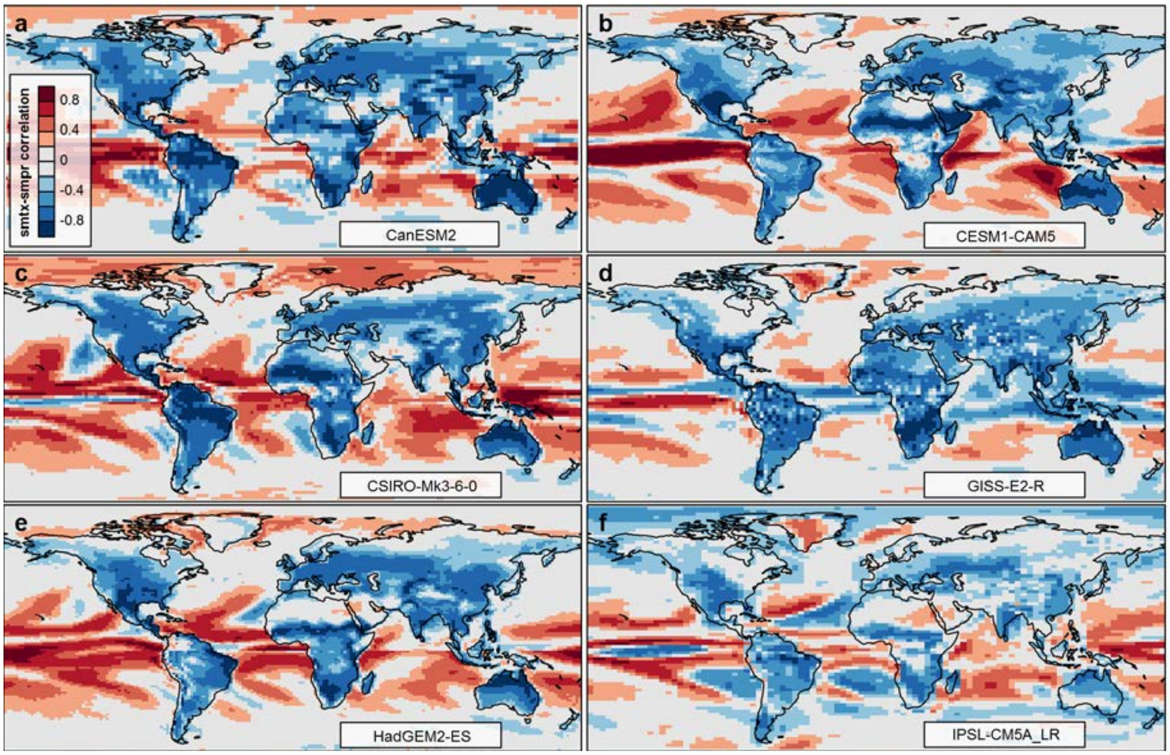

Supplementary Figure 2 | Correlation between summer precipitation (smpr) and mean daily maximum temperature in the summer (smtx) in the pooled historicalNat runs of the six CMIP5 models (panels a-f) analysed in this study.

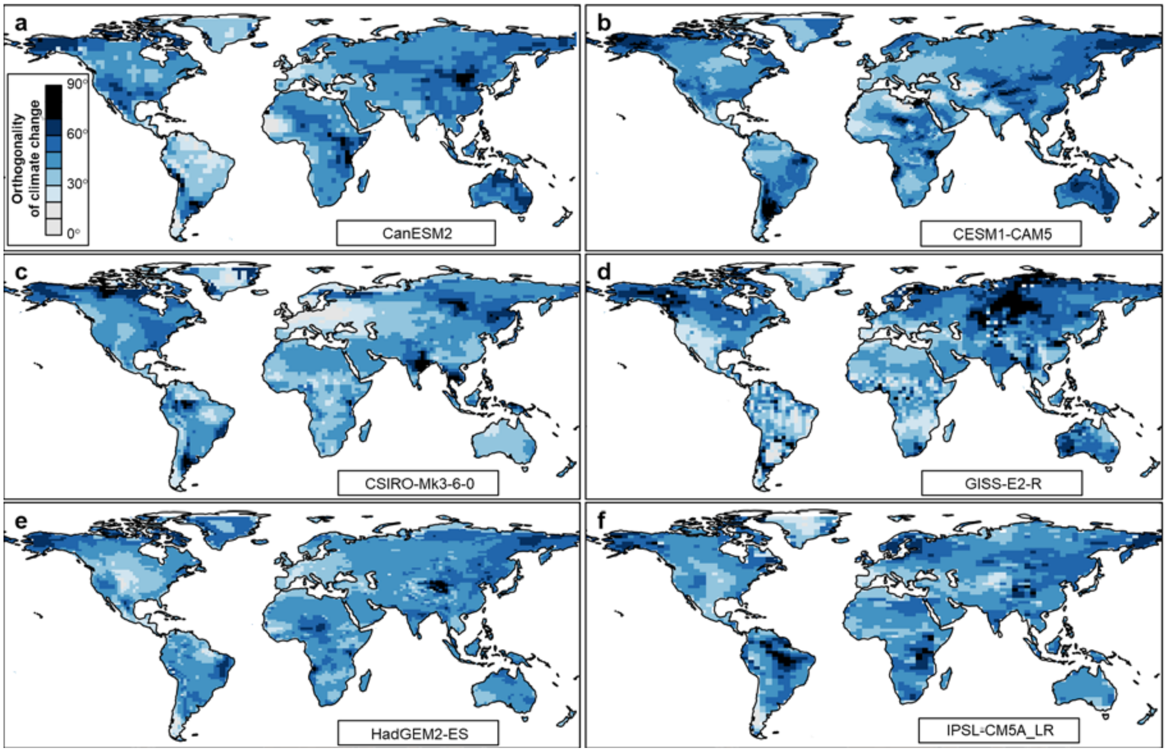

Supplementary Figure 3 | Orthogonality of climate change projected by the six CMIP5 models (panels a-f). Orthogonality of climate change is the arctan of the relative magnitude of the 2051-2100 normals in PC1 and PC2 of reference variability, providing degrees of angular displacement from the dominant axis of interannual variability.

Supplementary Note 3: Timing of climate departures

A major stream of the climate change detection literature has focused on the timing of the emergence of the climate signal from the noise of natural or historical variability. For most purposes, the time of emergence and the time of departure are equivalent concepts, and we treat them as such here. In the absence of a specific biological response, the specific metric and threshold for defining the departure year is arbitrary, and there are many such definitions in the literature. Here, we define a departure threshold as a 2σ proportion of 0.25; i.e., the climate is said to have departed from natural variability when 25% of the anomalies in the preceding 30-yr period are 2σ extremes or greater. Since the one-tailed null probability of a 2σ anomaly is 2.3%, this threshold approximates an order-of-magnitude increase in the frequency of warm 2σ anomalies. The relative timing of departure is the number of years that the bivariate signal departs prior to the departure of the univariate climate signal (max. of Pr or Tx). Locations at which the univariate signal has not departed by the year 2100 are assigned a departure year of 2100; this produces a conservative estimate of the departure of the bivariate climate signal relative to the univariate climate signal.

As expected, there is a strong relationship between the relative timing and the relative magnitude of departure (Supplementary Figure **4**). On average across all of the models, the bivariate climate signal departs more than 10 years prior to the univariate climate signal in 17% of land area (intermodel range of 7-23%). If the 2σ proportion threshold for departure were increased from 0.25 to 0.5, the relative timing of departure would increase in some areas, but would decrease or be undetected in others because the univariate departure year occurs beyond the year 2100 (manuscript Figure 2). The relative timing of departure is negative in some locations where the maximum departure difference is low. This occurs because the bivariate climate signal is weaker than the univariate signal at low correlations (see Supplementary Note 7).

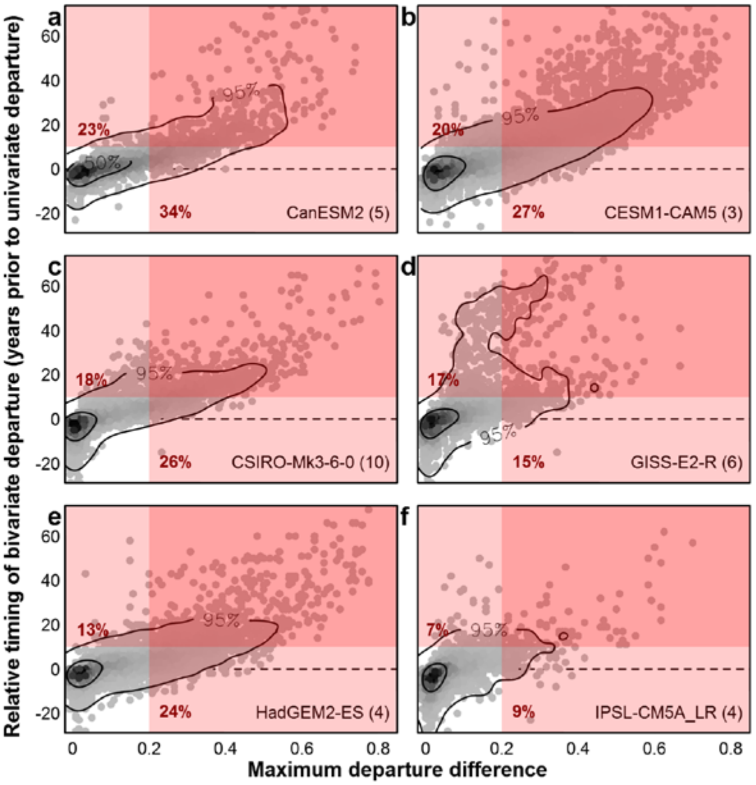

Supplementary Figure 4 | Relationship of the relative timing and the relative magnitude of climate departures in RCP4.5 ensemble projections of six CMIP5 models (panels a-f). Positive values on both axes indicate a bivariate (summer Tx & Pr) climate signal that is stronger (x-axis) and earlier (y-axis) than the univariate (max of Tx or Pr) climate signal. The climate signal is the average 2σ ratio of a number of model runs, given in parentheses next to the model name. Percentages in red are the proportion of the land area in each model with a maximum departure difference >0.2 or a relative departure timing of >10 years.

The bivariate summer Pr-Tx signal crosses the 2σ-proportion

threshold of 0.25 for climate departure as early as 1980 in the tropical regions of some models and as late as 2020 in others (Supplementary Figure 5). The spatial pattern of departure year is generally consistent with other studies of the time of emergence of the mean summer temperature signal [1,2]: departure year increases with latitude, though in some models the high Arctic experiences early departure. Many regions with strong departure intensification (manuscript Figure 3), e.g. the SE USA in CanESM2, do not show pronounced early relative timing of departure (Supplementary Figure 6). This occurs because the bivariate and univariate climate signals are only beginning to diverge when they cross the 2σ-proportion threshold of 0.25 for climate departure (manuscript Figure 2b).

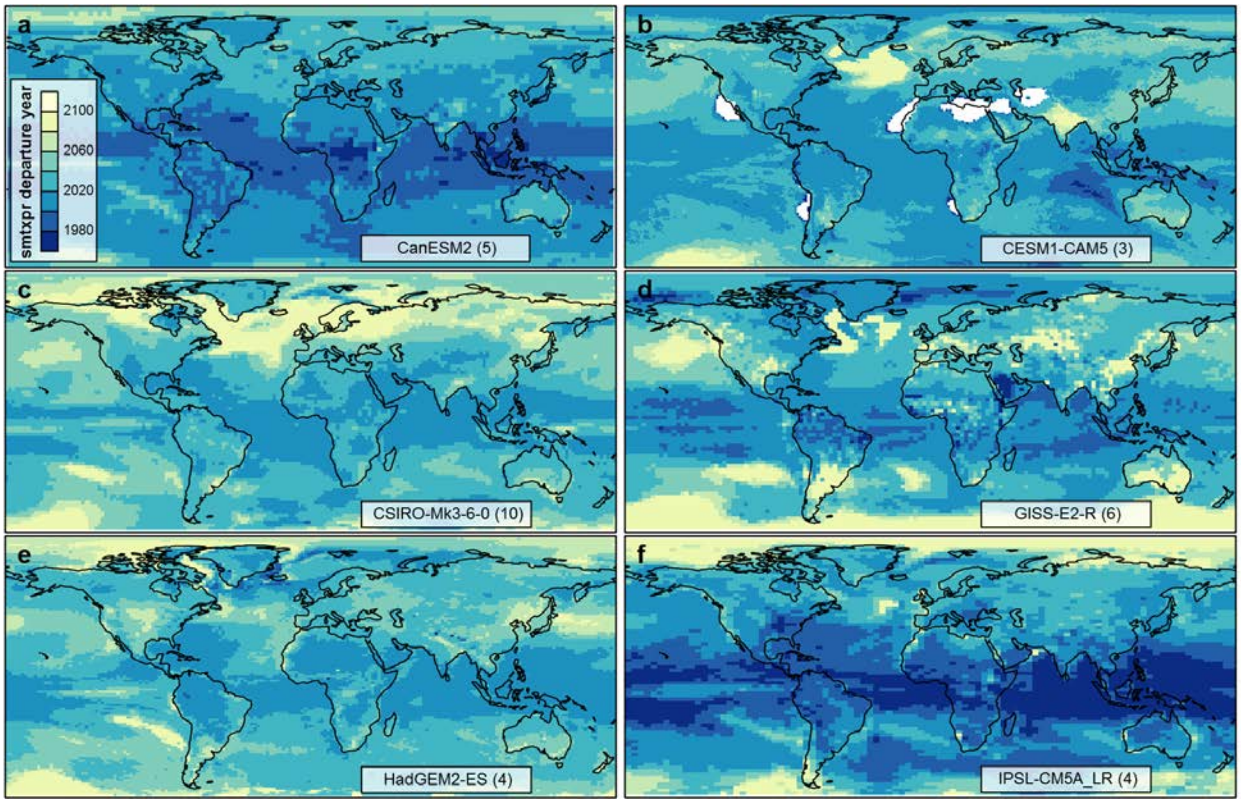

Supplementary Figure 5 | Departure year of the bivariate summer Tx-Pr climate signal from natural variability in the six CMIP5 models analysed in this study. Panels a-f show single-model ensemble results for each CMIP5 model. The number of runs in each ensemble is given in parentheses next to the model name.

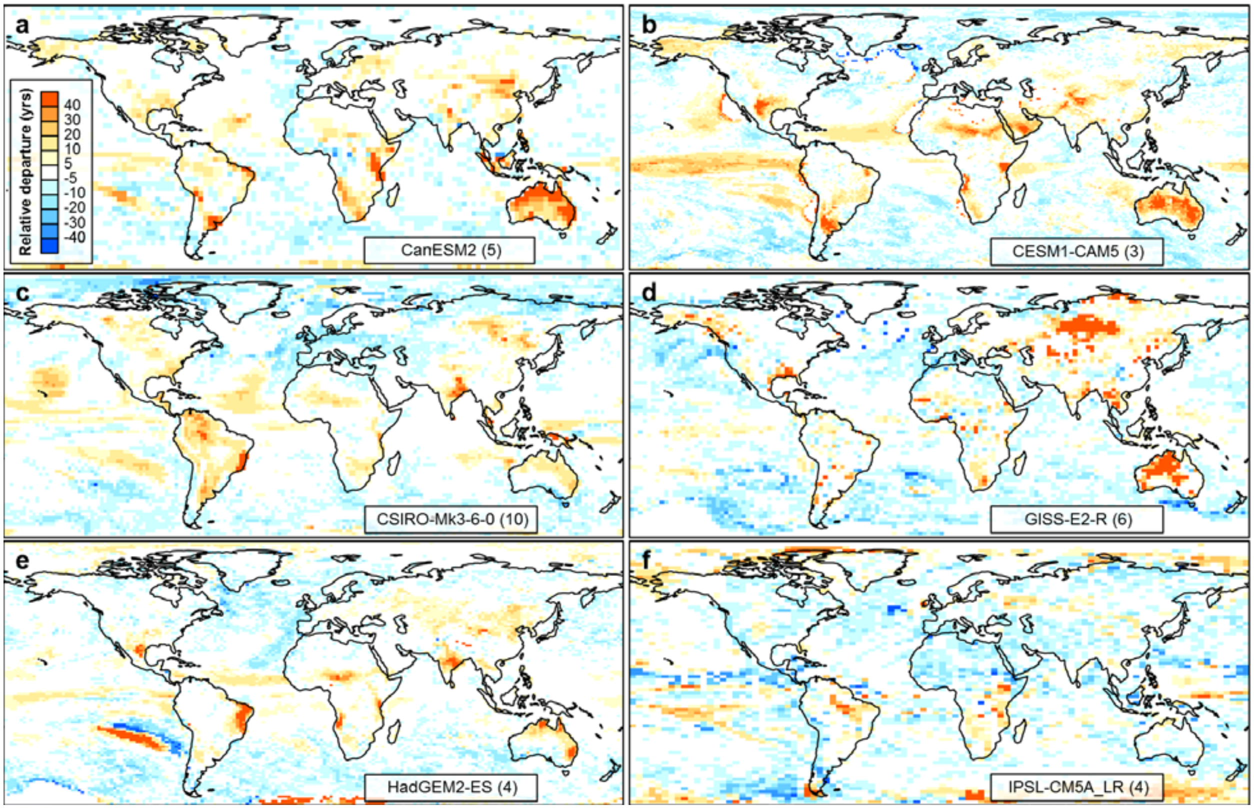

Supplementary Figure 6 | Timing of departure of the bivariate summer Tx-Pr climate signal relative to the departure of the univariate (max. of Tx or Pr) climate signals. Panels a-f show single-model ensemble results for each CMIP5 model. The number of runs in each ensemble is given in parentheses next to the model name. Positive numbers indicate the number of years that the bivariate signal departs prior to the univariate signal.

Supplementary Note 4. Parallel analysis of hottest three consecutive months

We used Boreal and Austral summer (JJA and DJF, respectively) in our main analysis to facilitate comparisons to other papers that use this common definition of summer, in particular the analysis of summertime temperature-precipitation correlations by Berg et al. (2015). However, we acknowledge that this definition of summer is problematic in the tropics and some subtropical regions, and confounds comparison with other relevant studies (e.g., Zscheischler and Seneviratne 2017). This section provides a parallel analysis which defines summertime as the hottest three consecutive months. This season is identified in each CMIP5 model from the mean monthly Tmax in the r1i1p1 historicalNat run (Supplementary Figure 7).

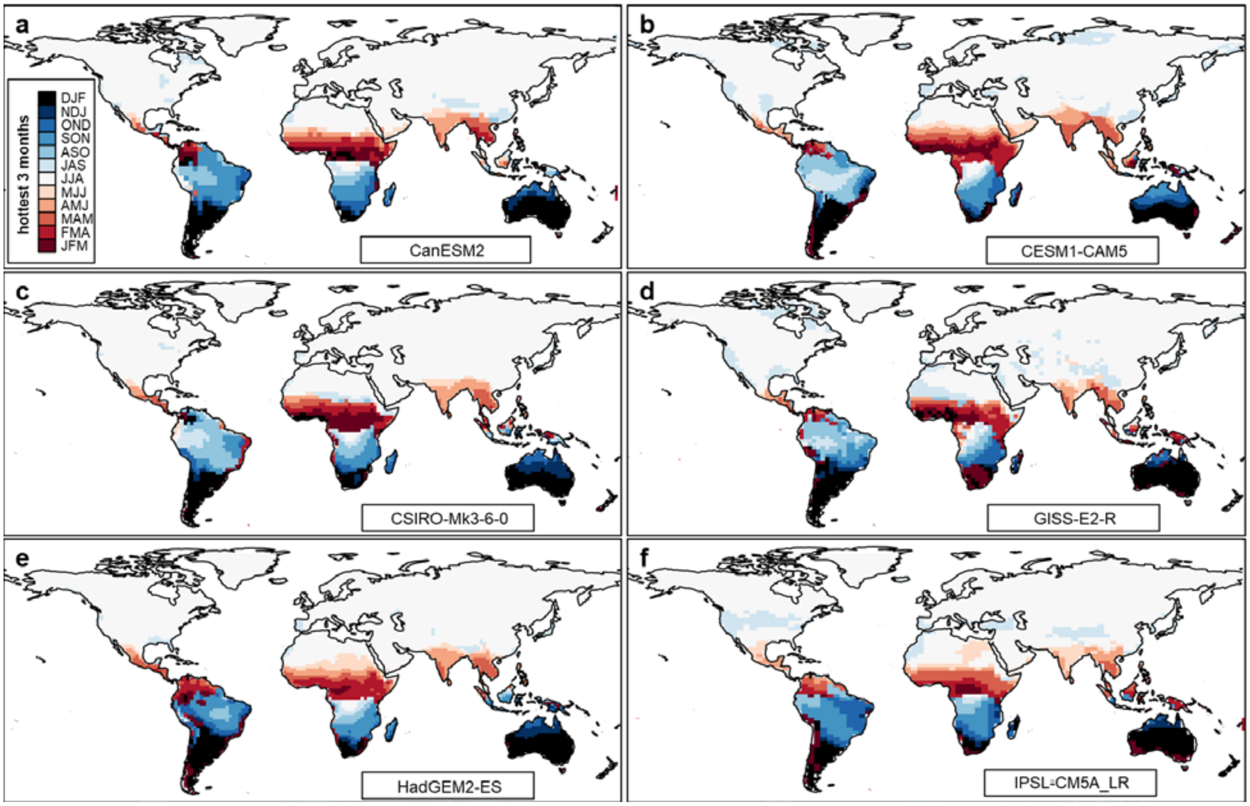

Supplementary Figure 7 | Hottest three consecutive months in the r1i1p1 historicalNat run of each CMIP5 model used in this analysis. Panels a-f show single-model ensemble results for each CMIP5 model. The number of runs in each ensemble is given in parentheses next to the model name.

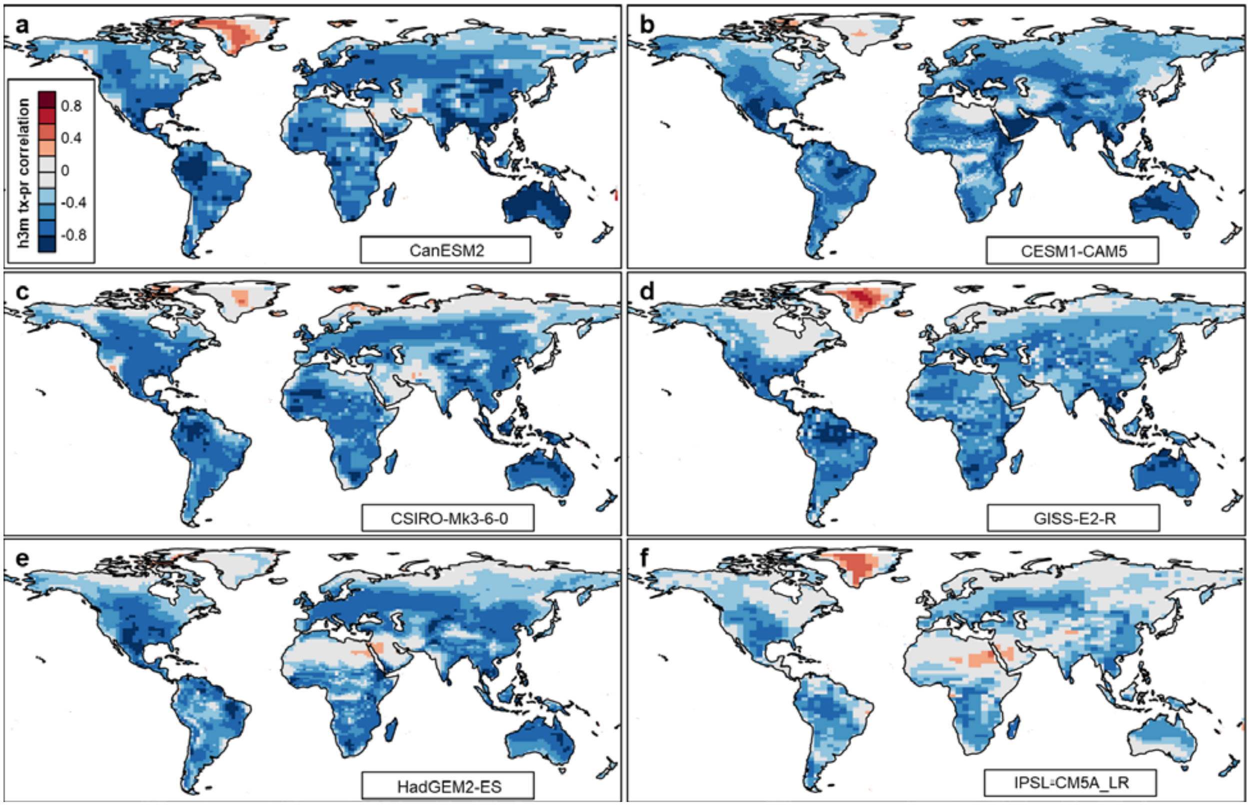

Supplementary Figure 8 | Correlation between precipitation (pr) and mean daily maximum temperature (tx) of the hottest 3 consecutive months (h3m) in the pooled historicalNat runs of the six CMIP5 models analysed in this study. Panels a-f show single-model ensemble results for each CMIP5 model. The number of runs in each ensemble is given in parentheses next to the model name.

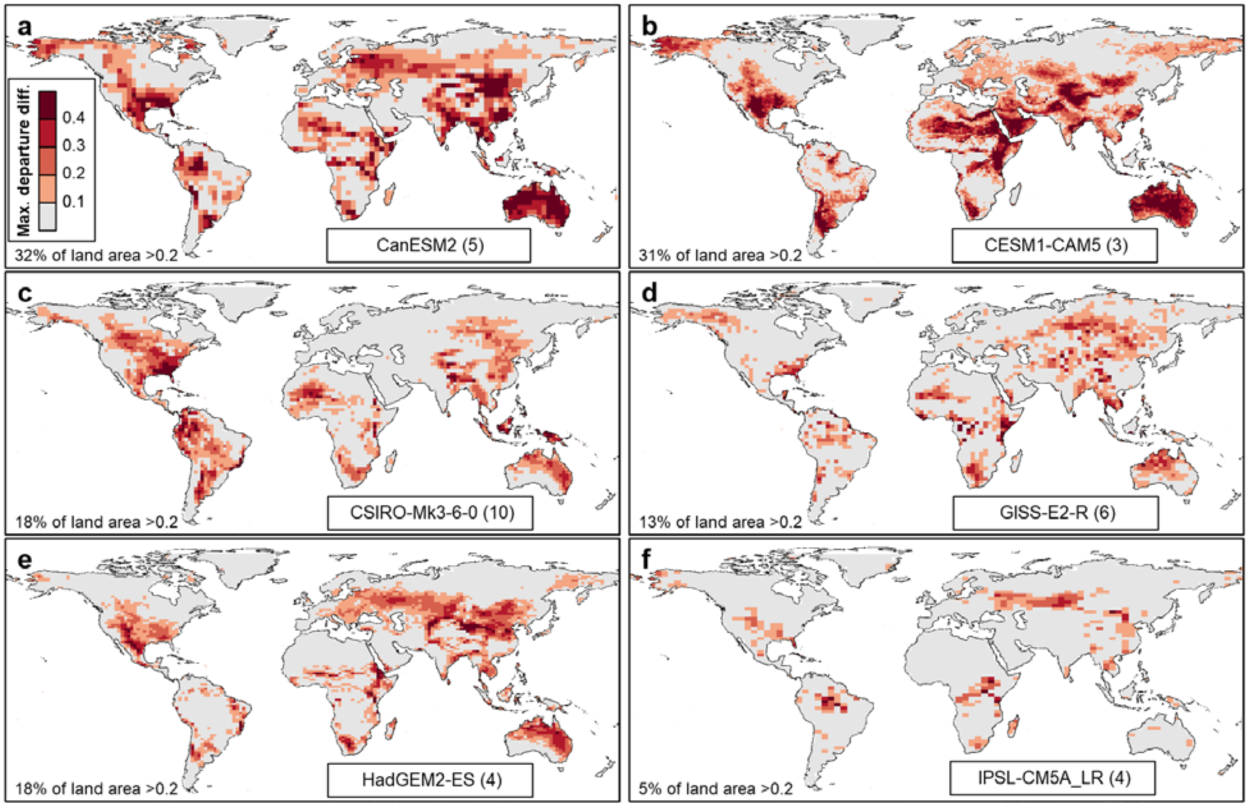

Supplementary Figure 9 | Intermodel variation in relative departures from natural variability in temperature (Tx) and precipitation (Pr) of the hottest three consecutive months. Panels a-f show single-model ensemble results for each CMIP5 model. The number of runs in each ensemble is given in parentheses next to the model name.

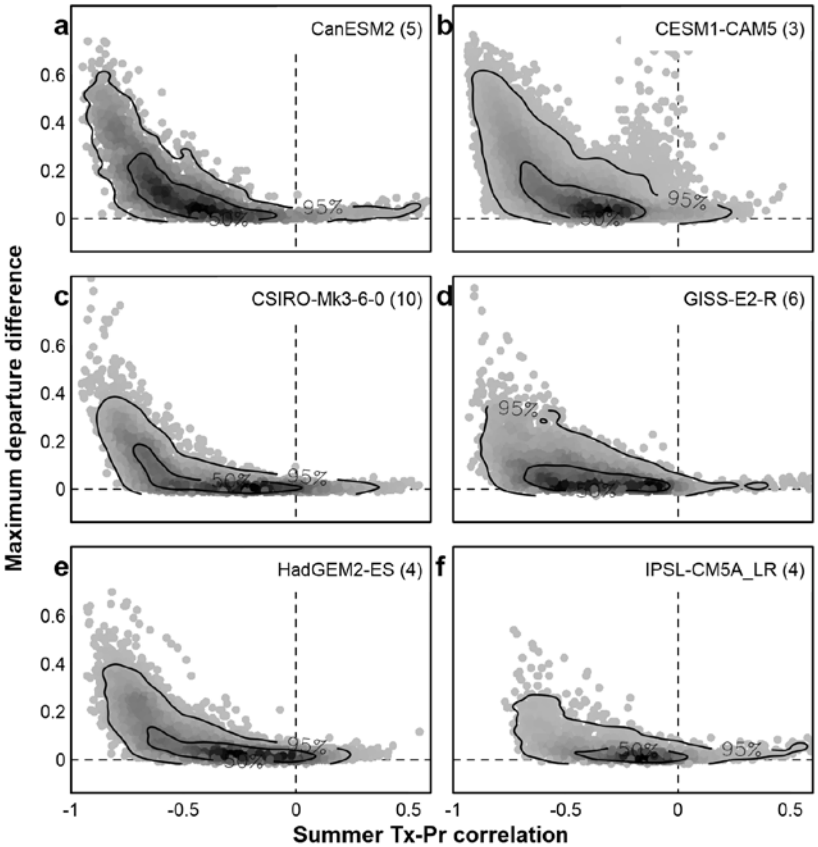

Supplementary Figure 10 | Relationship of maximum departure difference to the correlation between mean daily maximum temperature (Tx) and precipitation (Pr) of the three hottest consecutive months in RCP4.5 ensemble projections of 6 CMIP5 models. Panels a-f show single-model ensemble results for each CMIP5 model. The number of runs in each ensemble is given in parentheses next to the model name. Oceans and Antarctica are not plotted.

Supplementary Note 5: Out-of-reference-period standardized anomaly bias

The conventional method of calculating standardized anomalies uses the mean and standard deviation of variability during a reference period to assign sigma levels to observations. This method systematically overestimates the frequency of extreme events outside of the reference period because variation in the sample mean and variance reduction in the sample variance both increase the variance of out-of-sample standardized anomalies [5]. For small reference samples, this bias has a substantial impact on the detection of extreme observations: for a 30-"year" reference sample drawn from a normal distribution, the frequency of 2σ events in the out-of-sample period is overestimated by 29% (Supplementary Figure 11). In real observations, this bias can be expected to be greater because serial autocorrelation reduces the effective sample size of the reference period [5]. This bias is directly relevant to our study, which uses the frequency of 2σ events as its primary metric.

The reference period bias can be removed by using the student's t distribution instead of the normal distribution for inferring the probability (sigma level) of anomalies. We have not implemented this correction to the calculation of sigma dissimilarity in our study. However, we note that the bias associated with our very large pooled historicalNat reference periods is negligible (Supplementary Figure 11); simulation indicates that the bias in the 2σ ratio is 2% beyond n_{ref} = 465 years (the minimum n_{ref} in our study).

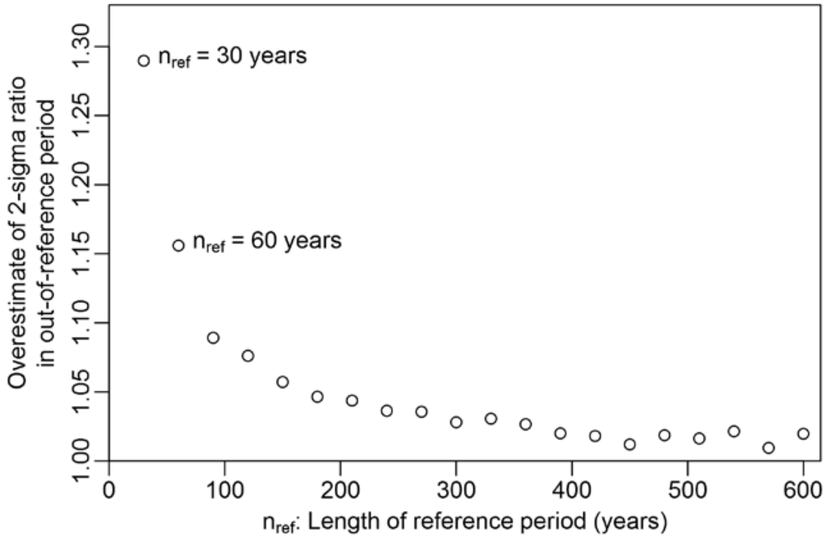

Supplementary Figure 11 | Overestimation bias in the 2σ proportion of non-reference-period standardized anomalies at reference period samples of nref = 30 to 600 normal variates ("years"). The bias at each nref is the mean bias from 10000 simulations, each with out-of-reference-period samples of n= 250 normal variates.

Supplementary Note 6: Sensitivity to normalization methods

Sigma dissimilarity assigns probabilities to Mahalanobis distances based on the assumption that reference period variability is multivariate normal (MVN). Violations of this assumption cause the probability of some anomalies to be underestimated and others to be overestimated. Despite univariate normalization, historicalNat Pr-Tx distributions are qualitatively non-MVN in some locations of the CanESM2 model. This likely is also the case in the other CMIP5 ensemble models. In this section we test the sensitivity of our results to one alternate method of univariate normalization and two methods of multivariate normalization. Based on this sensitivity analysis, we chose not to conduct multivariate normalization in order to maintain the simplicity and transparency of our primary methods.

We used the following methods for sensitivity analysis:

Univariate normalization only:

1. **qdm**: **quantile delta mapping** + sigma dissimilarity. This form of non-parametric quantile mapping [6] is used in the main text of our manuscript. The reference variability is normalized non-parametrically while preserving the magnitude of the climate change signal.
2. **pqm**: **parametric quantile mapping** + sigma dissimilarity. Fit a generalized gamma distribution to the reference variability and match the quantiles of the fitted distribution with the quantiles of the standard normal distribution. Parametric quantile mapping is commonly applied in the calculation of the standardized precipitation index [7].

Univariate normalization plus multivariate normalization:

3. **mbcn**: **multivariate quantile mapping** + sigma dissimilarity. This multivariate generalization of quantile delta mapping [8] is used to map the bivariate distribution of the historicalNat reference variability onto a standard bivariate normal distribution while preserving changes in the quantiles of each variable in the historical+RCP4.5 projection.
4. **kde**: quantile delta mapping + **bivariate kernel density estimation**. Instead of normalizing the bivariate distribution, this method uses kernel density estimation to map the probability contours of the bivariate distribution of reference period variability, allowing non-parametric estimation of the probability of bivariate anomalies.

These methods produce subtle but discernable differences in the magnitude, though not the spatial pattern, of maximum departure difference (Supplementary Figure **12**). Kernel density estimation produces reduced departure differences in all cells. This suggests that the other methods may be overestimating the univariate anomalies, since kde provides probability density estimates that are relatively independent of variable scaling. There are no discernible differences in the mbcn method relative to qdm, indicating either that non-normality is not an important factor in departure difference in the CanESM2 model, or that mbcn is not an effective multivariate normalization method for this particular application. Despite these sensitivities, the occurrence and the spatial pattern of departure intensification is robust to the differences in the four methods tested here.

The 2σ proportion underlies the observed robustness of departure intensification to normalization methods. As a binary metric, the 2σ proportion is unaffected by the effect of normalization on the magnitude of anomalies beyond the 2σ threshold. The 2σ proportion only relies on statistical inferences that are strongly supported by the reference variability sample. This is a critical advantage over the signal-to-noise ratio (z-scores of reference variability).

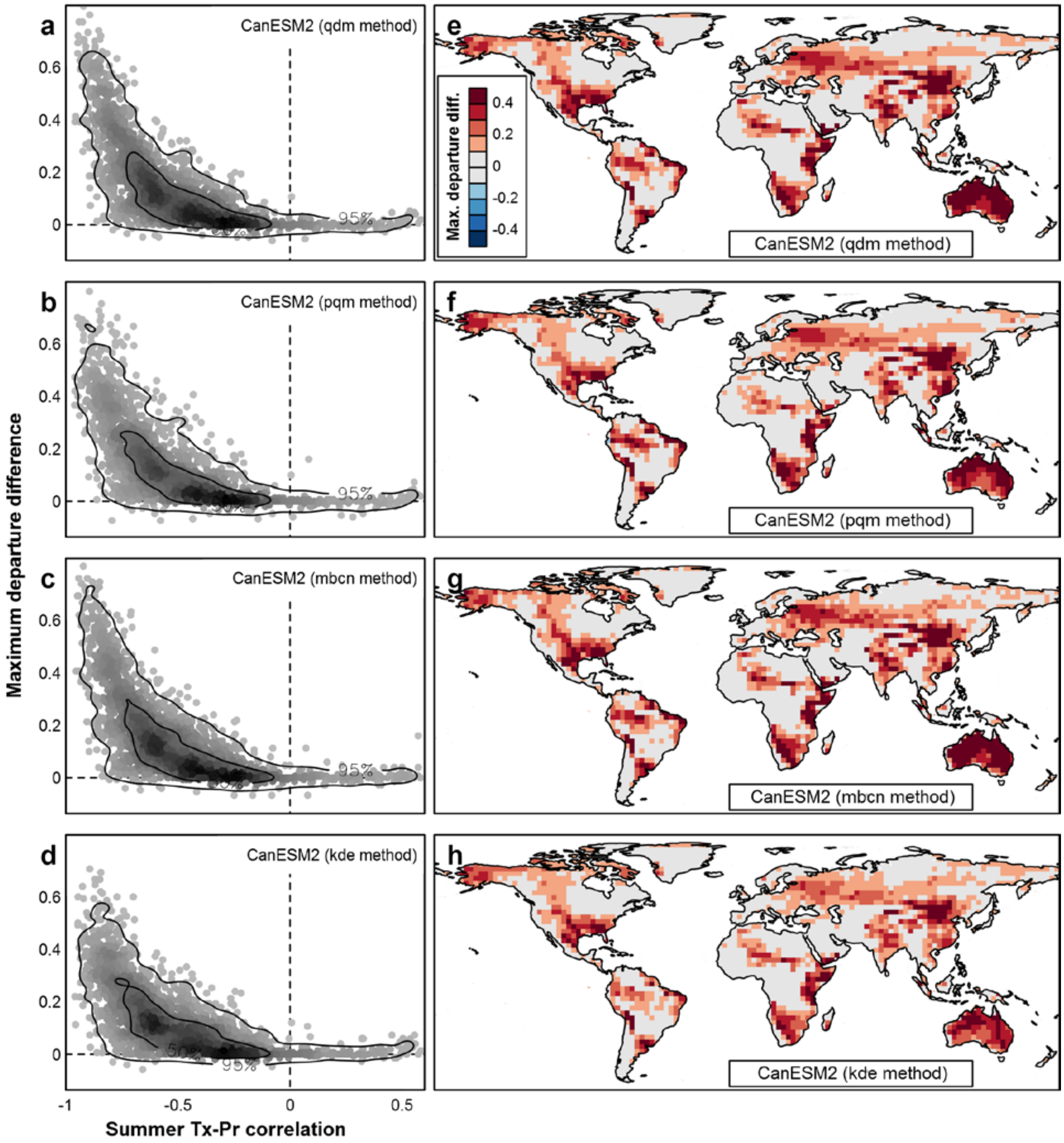

Supplementary Figure 12 | Sensitivity of maximum departure difference to four different treatments of multivariate normality: **a,e,** quantile delta mapping (qdm); **b,f,** parametric quantile mapping (pqm); **c,g,** multivariate quantile mapping (mbcn); and **d,h,** bivariate kernel density estimation (kde). Results are shown for the CanESM2 Historical + RCP4.5 ensemble, consisting of 5 projections of the 1850-2100 period.

Supplementary Note 7: Null model for departure differences

Departure difference can be positive or negative, as seen in maps for the 2021-2050 period (Supplementary Figure **13**). Departure difference is negative in cases where there is little trend in precipitation and low correlation between temperature and precipitation. Given that the signal-to-noise ratio of precipitation is generally much less than temperature, the scenario of non-stationary temperature, stationary precipitation, and a zero correlation is an appropriate null model for departure difference.

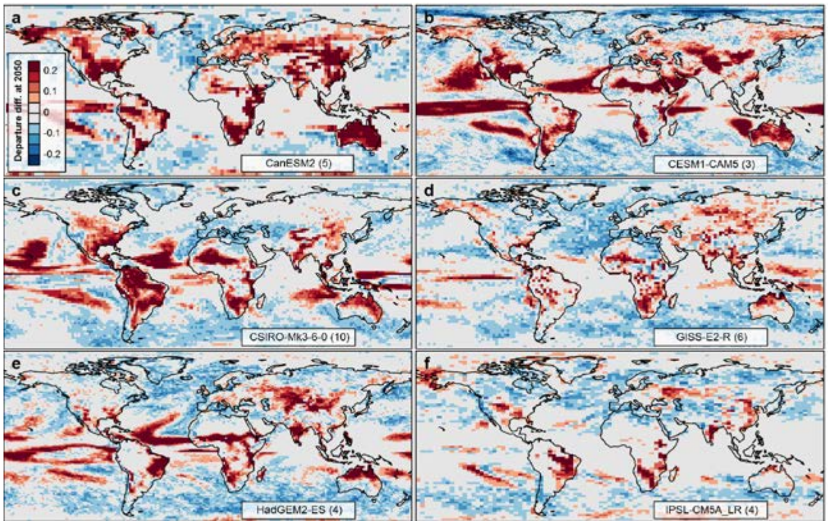

Supplementary Figure 13 | Departure differences in the CMIP5 ensemble during the 2021-2050 period. Panels a-f show single-model ensemble results for each CMIP5 model. The number of runs in each ensemble is given in parentheses next to the model name.

A simulation of two normal variables, *x* with a shifted mean (representing a non-stationary Tx signal) and *y* with a mean of zero (representing a stationary Pr signal). As the mean of *x* increases, the frequency of 2σ anomalies increases faster for *x* alone than for the bivariate distribution of *x* and *y* (Supplementary Figure 14). This occurs because the probability of a distance of 2 is lower for a bivariate normal distribution than for a univariate normal distribution (see Figure 6 in the manuscript). For a 2σ shift in the mean of *x*, the bivariate frequency of 2σ anomalies is 0.1 less than the univariate frequency.

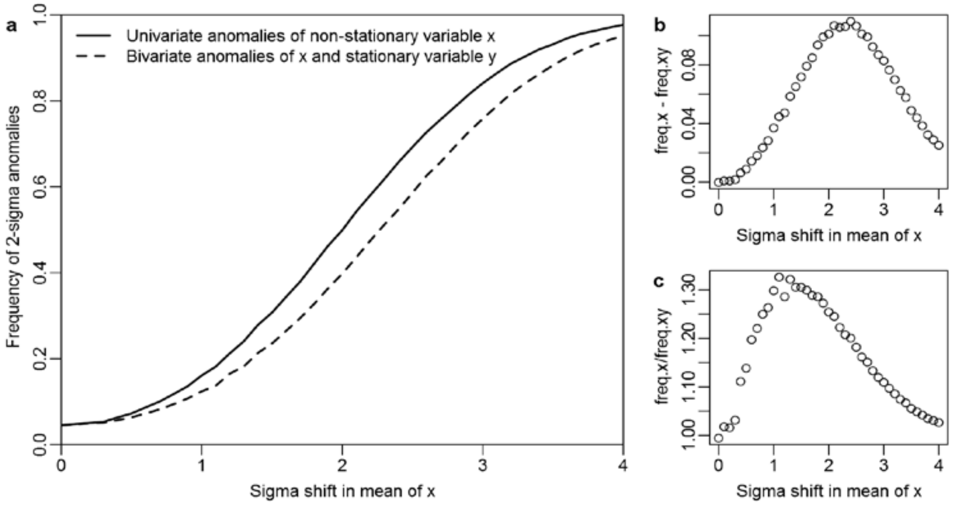

Supplementary Figure 14 | Null model for departure differences under global warming. **a**, Mean 2σ proportion of 10000 samples of two normal variables, *x* with a shifting mean and *y* with a mean of zero. b, Departure difference and departure ratio of univariate x anomalies and bivariate x-y anomalies.

Supplementary Note 8: Pseudocode for calculation of maximum departure difference

The departure difference is calculated with the following steps:

1. For each cell in the grid of each CMIP5 model,
2. Start with [n,k] matrices X_{Tx}, X_{Pr}, and X_{Bi} of standardized anomalies for the T_{max}, precipitation, and bivariate time series, respectively. Each of the n rows is a year in the annual time series 1850-2100, and each of the k columns is a historical+RCP4.5 run.
3. For each year 1880-2100 and each model run, divide the number of absolute values exceeding 2 (2σ exceedances) in the previous thirty years by thirty, producing [n-30,k] matrices Y_{Tx}, Y_{Pr}, and Y_{Bi}, of 2σ proportions for the T_{max}, precipitation, and bivariate time series, respectively.
4. For each element of Y_{Tx} and Y_{Pr}, i.e., each year of each model run, select the 2σ proportion for either Y_{Tx} and Y_{Pr}, whichever is greater, producing an [n-30,k] matrix Y_{Uni} of univariate 2σ proportions.
5. For each year 1880-2100, calculate the mean of the 2sigma proportions across all model runs, producing one univariate time series, $\overline{Y_{Uni}}$ and one bivariate time series $\overline{Y_{Bi}}$.
6. The departure difference is an 1880-2100 time series of bivariate minus univariate 2sigma proportion, i.e., $\overline{Y_{Uni}}$ minus $\overline{Y_{Bi}}$.
7. The maximum departure difference is the maximum value in the departure difference time series.

An alternative approach would be to calculate maximum departure difference with respect to each variable separately ($\overline{Y_{Tx}}$ and $\overline{Y_{Pr}}$), and select the minimum of these two values for each cell. The sensitivity of the results to this valid alternative approach are likely very low because the univariate departures are almost exclusively driven by the Tx climate change trend (see Supplementary Note 1)

Supplementary Tables

Model	Modeling Center (or Group)	Institute ID	lat resolution	lon resolution	TCR	ECS	histNat runs	hist+RCP4.5 runs
CanESM2	Canadian Centre for Climate Modelling and Analysis	CCCMA	2.8	2.8	2.4	3.7	5	5
CESM1-CAM5	Community Earth System Model Contributors	NSF-DOE-NCAR	0.9	1.3	2.3	4.1	3	3
CSIRO-Mk3-6-0	Commonwealth Scientific and Industrial Research Organization in collaboration with Queensland Climate Change Centre of Excellence	CSIRO-QCCCE	1.9	1.9	1.8	4.1	5	10
GISS-E2-R	NASA Goddard Institute for Space Studies	NASA GISS	2.0	2.5	1.5	2.1	5	6
HadGEM2-ES	Met Office Hadley Centre (additional HadGEM2-ES realizations contributed by Instituto Nacional de Pesquisas Espaciais)	MOHC	1.3	1.9	2.5	4.6	4	4
IPSL-CM5A_LR	Institut Pierre-Simon Laplace	IPSL	1.9	3.8	2	4.1	3	4

Supplementary Table 1 | CMIP5 models included in this study. The number of historicalNat runs and historical + RCP4.5 runs used in our analyses are specified. Perturbed physics experiments are excluded. CMIP5 is described by Taylor et al. (2012). Adjusted transient climate response (TCR) and equilibrium climate sensitivity (ECS) values are reported by Forster et al. (2013), except for non-adjusted values for CESM1-CAM5 reported by Meehl et al. (2013).

Supplementary References

1. Mahlstein, I., Knutti, R., Solomon, S. & Portmann, R. W. Early onset of significant local warming in low latitude countries. *Environ. Res. Lett.* **6,** 34009 (2011).
2. Hawkins, E. & Sutton, R. Time of emergence of climate signals. *Geophys. Res. Lett.* **39,** 1–6 (2012).
3. Berg, A. *et al.* Interannual coupling between summertime surface temperature and precipitation over land: Processes and implications for climate change. *J. Clim.* **28,** 1308–1328 (2015).
4. Zscheischler, J. & Seneviratne, S. I. Dependence of drivers affects risks associated with compound events. *Sci. Adv.* **3,** e1700263 (2017).
5. Sippel, S. *et al.* Quantifying changes in climate variability and extremes: Pitfalls and their overcoming. *Geophys. Res. Lett.* **42,** 9990–9998 (2015).
6. Cannon, A. J., Sobie, S. R. & Murdock, T. Q. Bias correction of GCM precipitation by quantile mapping: How well do methods preserve changes in quantiles and extremes? *J. Clim.* **28,** 6938–6959 (2015).
7. Guttman, N. B. Accepting the standardized precipitation index: a calculation algorithm. *J. Am. Water Resour. Assoc.* **35,** 311–322 (1999).
8. Cannon, A. J. Multivariate quantile mapping bias correction: an N-dimensional probability density function transform for climate model simulations of multiple variables. *Clim. Dyn.* (2017). doi:10.1007/s00382-017-3580-6
9. Forster, P. M. *et al.* Evaluating adjusted forcing and model spread for historical and future scenarios in the CMIP5 generation of climate models. *J. Geophys. Res. Atmos.* **118,** 1139–1150 (2013).
10. Meehl, G. A. *et al.* Climate change projections in CESM1(CAM5) compared to CCSM4. *J. Clim.* **26,** 6287–6308 (2013).
11. Taylor, K. E., Stouffer, R. J. & Meehl, G. A. An overview of CMIP5 and the experiment design. *Bull. Am. Meteorol. Soc.* **93,** 485–498 (2012).
