# Supplementary material for: Wetter summers can intensify departures from natural variability in a warming climate
Source: Nat Commun. 2018 Feb 22;9:783. doi: 10.1038/s41467-018-03132-z (PMC5823852; doi:10.1038/s41467-018-03132-z)
Supplement: Supplementary file 3 — Description of Additional Supplementary Files [file 41467_2018_3132_MOESM3_ESM.docx]

**Description of Additional Supplementary Files**

File Name: Supplementary Data 1

Description: A subset of the CanESM2 global climate model grid data for use with the R code provided.

File Name: Supplementary Software 1

Description: R code demonstrating the core analytical and graphical methods of the manuscript.
